# Cyclic Peptides: Promising Scaffolds for Biopharmaceuticals

**DOI:** 10.3390/genes9110557

**Published:** 2018-11-16

**Authors:** Donghyeok Gang, Do Wook Kim, Hee-Sung Park

**Affiliations:** Department of Chemistry, Korea Advanced Institute of Science and Technology, 291 Daehak-ro, Yuseong-gu, Daejeon 34141, Korea; gangdh0154@kaist.ac.kr (D.G.); toyeca@kaist.ac.kr (D.W.K.)

**Keywords:** cyclic peptides, biopharmaceuticals, mRNA display, yeast two hybrid

## Abstract

To date, small molecules and macromolecules, including antibodies, have been the most pursued substances in drug screening and development efforts. Despite numerous favorable features as a drug, these molecules still have limitations and are not complementary in many regards. Recently, peptide-based chemical structures that lie between these two categories in terms of both structural and functional properties have gained increasing attention as potential alternatives. In particular, peptides in a circular form provide a promising scaffold for the development of a novel drug class owing to their adjustable and expandable ability to bind a wide range of target molecules. In this review, we discuss recent progress in methodologies for peptide cyclization and screening and use of bioactive cyclic peptides in various applications.

## 1. Introduction

Screening and identification of chemicals or molecules that can bind to or inhibit particular cellular targets is a critical step in biomedical and related research. For decades, natural compounds and man-made molecules have been searched for and exploited for this purpose. These molecules can be broadly classified into two categories: small molecules and macromolecules.

Small molecules have a molecular weight less than 1 kDa and are generally hydrophobic. This latter property enables small molecules to easily pass through the cell membrane and diffuse across intracellular space—and throughout the whole body—thus allowing for oral administration. Small molecules can be chemically synthesized, making mass production possible and quality control manageable. Despite such advantages and long tradition of use, small molecules still have some unresolved intrinsic limitations. Because of their small size and high hydrophobicity, small molecules can only interact with targets that have a rigid and hydrophobic patch or groove. In addition, small molecule binding regions or features are highly conserved among various cellular proteins that are closely or distantly related to target proteins [1]. For example, the ATP-binding domain has been a prime binding site for small molecules, but it is well conserved among protein kinases. Accordingly, many kinase inhibitors suffer from low selectivity, even after they have been screened and evolved for specific targets [2]. This low selectivity can inevitably cause many unexpected side effects.

Macromolecules have a wide range of molecular weights, from 5 kDa to 150 kDa [3,4]. Many are amino acid-based polypeptides, and some contain nucleic acids. Because of their large contact area and hydrophilic regions, which allow multiple tight interactions with a target, macromolecules usually have strong binding ability and are highly selective for their target. Such favorable characteristics, which small molecules lack, have expedited the development of macromolecule-based drugs; many macromolecules, mostly antibodies, have been approved and are now available commercially. However, macromolecules suffer from their own critical problems that have yet to be overcome. For example, they are intrinsically unable to pass through cellular membranes; although their membrane transport can be improved by many means, such as conjugating to cell-penetrating peptides, success is limited and efficiency of transport is low [5,6]. Thus, their use is largely restricted to extracellular targets. Moreover, most macromolecules are currently produced using cell culture methods; thus, heterogeneity owing to post-translational modifications and other derivatizations are inevitable. Lastly, they are not orally available, and must generally be administered intravenously. Thus, macromolecules are easily degraded and neutralized in the bloodstream and can even cause severe immunogenicity [7].

Cyclic peptides have gained increasing attention in recent years as an alternative scaffold. Cyclic peptides are usually composed of 5 to 14 amino acids and have a molecular weight of about 500 to 2000 Da. The moderate size and diverse functional groups of peptides ensure that the contact area is large enough to provide high selectivity, and their potential to form multiple hydrogen bonds can lead to strong binding affinity. In addition, cyclization of peptides generates structural and functional features that are critical for their use as pharmaceutical agents [8]. The structural constraints provided by cyclization help to resist degradation by proteases in the blood, thereby increasing their serum stability [9]. Cyclization of peptides also facilitates passage through the cell membrane, thus broadening the potential use of cyclic peptides beyond extracellular targets to include intracellular targets [10]. Because of such favorable features, various cyclic peptides from natural sources and their derivatives have been exploited for biomedical and other purposes. Inspired by natural cyclic peptides, numerous approaches for designing man-made cyclic peptides with customized structural and functional properties have been in the spotlight in recent years. In this review, we will first discuss several representatives of natural cyclic peptides. Then, we will examine various methods that have been used for peptide cyclization, from ordinary chemical synthesis to enzymatic methods. Next, we will consider library construction and strategies for selecting active cyclic peptides. Lastly, potential applications of cyclic peptides in biomedical and other research areas will be described.

## 2. Natural Cyclic Peptides

**Figure 1:**
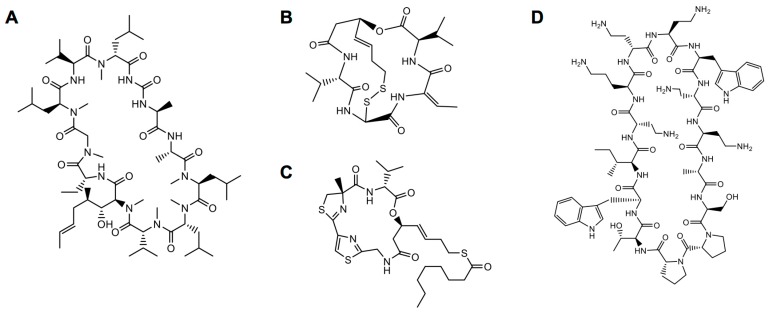
Natural cyclic peptides. (**A**) Cyclosporine A. (**B**) Romidepsin. (**C**) Largazole. (**D**) Murepavadin.

Cyclosporine A, a natural cyclic peptide composed of 11 amino acids, has been used since the early 1980s as an immunosuppressive drug (Figure 1A). This cyclic peptide binds to cyclophilin in T-cells, forming a cyclosporine A-cyclophilin complex that inhibits the calcium/calmodulin-dependent protein phosphatase, calcineurin. Calcineurin induces dephosphorylation and nuclear translocation of the transcription factor NF-AT (nuclear factor of activated T cells), which promotes transcription of interlukin-2 and some cytokine genes and causes activation of T cells. Cyclosporin A inhibits this phosphatase pathway, thereby suppressing the activity of T-cells and repressing the immune system [11].

Romidepsin, a natural cyclic depsipeptide from *Chromobacterium violaceum* contains one or more ester bonds in place of amide bonds (Figure 1B). This cyclic peptide has a disulfide bond between two internal sulfhydryls that is reduced upon entry of the peptide into the cell. Reduction of this disulfide bond yields two thiol groups linked to long alkyl chains that can chelate divalent metal ions, especially zinc ions in the active site of class I histone deacetylase (HDAC), thus inhibiting HDAC activity [12]. Histone deacetylase, an essential epigenetic controller, is often overexpressed in many cancers. By inhibiting HDACs, romidepsin can restore the expression of genes involved in apoptosis, ultimately repressing the proliferation and differentiation of cancer cells [13]. Romidepsin was approved by the Food and Drug Administration (FDA) for cutaneous T-cell lymphoma in 2009 and for peripheral T-cell lymphomas in 2011. Largazole, a natural cyclic peptide from the marine cyanobacteria *Symploca* sp., has similar HDAC inhibiting activity (Figure 1C). Analogous to romidepsin, largazole is a kind of prodrug in which one aliphatic carboxylic acid-protected thiol group forms a thioester. The protecting group is cleaved by enzymes after translocation of largazole into the cytoplasm, and the exposed thiol group combines with a zinc ion cofactor in HDAC to inhibit its activity [14].

Murepavadin is a synthetic cyclic peptidomimetic derived from the natural peptide protegrin-1 (PG-1), containing two d-amino acids and 12 l-form proteogenic/nonproteogenic amino acids (Figure 1D). Because of its l-proline-d-proline scaffold, this cyclic peptide has a stabilized β-hairpin conformation. Murepavadin targets lipopolysaccharide transport protein D (LptD) of *Pseudomonas aeruginosa*, blocking its transport function and causing alterations in lipopolysaccharide in the outer membrane of bacteria, leading to cell death. On the basis of such antimicrobial activity [15,16], murepavadin is currently in Phase III clinical trials for treatment of nosocomial pneumonia.

## 3. Generation of Synthetic Cyclic Peptides

Inspired by pharmaceutical applications of natural cyclic peptides, researchers have developed several strategies for creating synthetic cyclic peptides with tailor-made functionalities. Cyclization of peptide has been achieved using various methods, including scaffold-based and enzyme-based approaches. Scaffold-based cyclization employs reactions between chemical compounds and specific functional groups of amino acids, whereas enzyme-based cyclization exploits naturally existing enzymes that can cyclize their own substrates.

### 3.1. Scaffold-Based Cyclization

**Figure 2:**
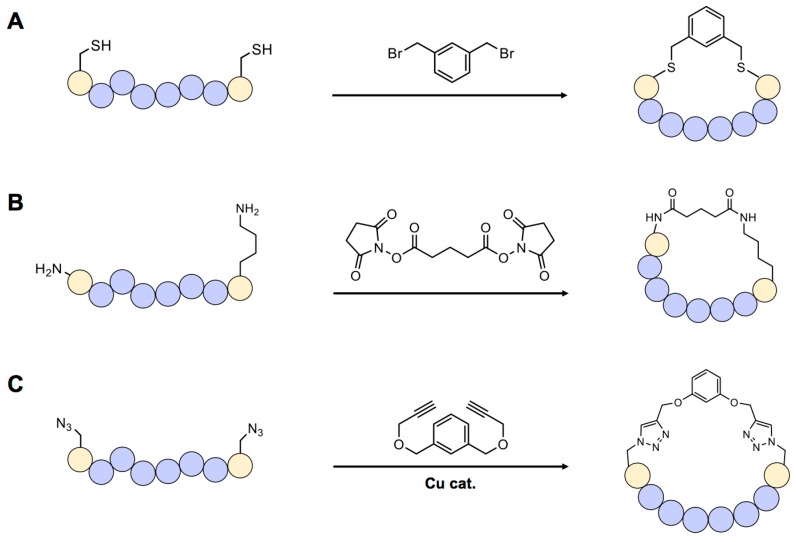
Scaffold-based cyclic peptide synthesis. (**A**) Organobromides selectively react with sulfhydryl group of cysteine. (**B**) *N*-hydroxysuccimide group reacts with the primary amine in p peptide and forms a stable amide bond. (**C**) Click reaction between azide and alkyne groups can be used for cyclization of chemically synthesized peptide.

Scaffold-based cyclization is one of the most frequently used methods because it can be applied to chemically or biologically synthesized peptides. In general, scaffold compounds such as organohalides (most frequently organobromides) selectively react with the sulfhydryl group of cysteine [17] (Figure 2A). One example of cysteine-specific scaffold-mediated cyclization includes modification of a peptide library displayed on bacteriophage [18]. In this approach, a library of genetically encoded peptides located between two cysteine residues can be transformed into circular forms by treating with the organobromide, tribromomethylbenzene (TBMB). These cyclic peptides can then be screened using the general phage-display method. Synthesis of double-bridged cyclic peptides using one or two kinds of organobromides has also recently been reported [19]. These di-bridged peptides were selected using the phage-display technique and the resulting peptide “hits” were found to have a binding affinity towards the target, plasma kallikrein, 10-times stronger than that of a previously screened bicyclic peptide.

Non-sulfhydryl groups, such as the primary amine of lysine or N-terminal amino group in a peptide, can be used for cyclization. *N*-hydroxysuccimide (NHS)-containing chemicals have long been used for this purpose (Figure 2B) [20], but this requires precise control of reaction conditions because NHS is easily hydrolyzed by water at physiological pH. Especially designed unnatural amino acids can be used for cyclization in peptides via a bio-orthogonal reaction. For example, if an azide-containing amino acid such as azidohomoalanine or azidophenylalanine exists in a peptide, a copper-mediated click reaction with an alkyne-bearing scaffold can lead to cyclization [21] (Figure 2C).

One of the greatest advantages of the scaffold-based method is the ease of controlling the cyclization process—and thus the size and hydrophilicity of the peptide—by changing the chemical structure of the scaffold. Multiple reaction sites, such as azide or alkyne groups, can be added to create further modifications, generating a multicyclic structure that is extremely rigid and stable under biological conditions. However, chemical scaffolds for cyclization may change the natural properties of peptides [22]. Moreover, in many peptide libraries, cysteines or lysines are present at multiple positions, potentially causing unpredictability and heterogeneity in the screening process.

### 3.2. Enzyme-Based Cyclization

**Figure 3:**
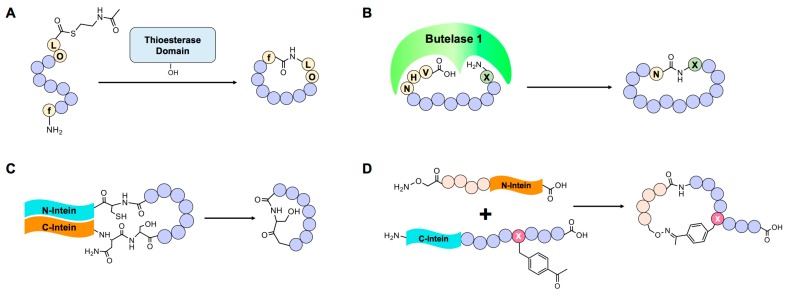
Enzyme-based cyclic peptide synthesis. (**A**) Cyclization of synthetic peptide using thioetsterase domain of nonribosomal peptide synthetase (NRPS). (**B**) Butelase 1-mediated cyclization requires C-terminal Asn-His-Val (NHV) tripeptide. After cyclization, only asparagine residue remains. (**C**) Split intein-mediated circular ligation for the synthesis of cyclic peptide. (**D**) Semi-synthetic method for peptide cyclization via oxime ligation using chemically synthesized N-intein and biologically prepared C-intein carrying the unnatural amino acid p-acetophenylalanine.

Many natural cyclic peptides are synthesized in a ribosome- and mRNA-independent manner by specialized nonribosomal peptide synthetase (NRPS) enzymes using various non-proteogenic amino acids, including D-amino acids [23]. The NRPS system is composed of several modules, each of which is divided into at least three basic domains: adenylation (A domain), transfer (T domain), and condensation (C domain). The A domain recognizes proteogenic and non-proteogenic amino acids and adenylates its substrate using ATP. Aminoacyl-AMP (AA-AMP), produced by the A domain, reacts with an alkyl thiol in the T domain to form an AA-T domain. Then, the Nth module’s C domain catalyzes formation of an amide bond between the N–1th and Nth amino acid. The last domain in the last module of NRPS is the termination (TE) domain, which determines the conformation of a synthesized peptide. If the thioester bond formed between the peptide and TE domain is cleaved by water or other nucleophiles, a linear peptide is released. However, if the thioester bond is attacked by an intramolecular nucleophile, such as an N-terminal amine, the primary amine of lysine, a serine hydroxyl group or a cysteine sulfhydryl group, a cyclic peptide is formed and released [24]. The chemical diversity of nonribosomal peptides can be expanded by changing the substrate specificity of the A domain or by replacing the whole A domain itself [25,26]. Such efforts have been successful in producing novel cyclic peptides, but the efficiency of the process is low [25]. To increase production efficiency, researchers developed a method that uses a synthetic non-native precursor peptide and thioester domain of NRPS (Figure 3A) [27,28]. Although the ratio of cyclic-to-linear forms of substrate peptide produced by this method was reduced, three different chemically prepared substrate peptides were successfully cyclized.

Another interesting example of cyclic peptide-containing non-proteogenic amino acids is cyanobactin [29]. Like the NRPS system, multiple genes are involved in the synthesis of cyanobactin. One major difference between nonribosomal peptides and cyanobactin is that the precursor of cyanobactin, composed of a leader peptide and core peptide, is encoded in the genome of the marine cyanobacteria and is translated by ribosomes. After translation of the precursor, enzymes in the same gene cluster recognize the leader peptide sequence and modify amino acids in the core region. The amino acid tolerance of these trunkamide-synthesizing enzymes was tested using a genetically encoded peptide library [30]. To determine whether non-native trunkamide peptides could be synthesized using this approach, researchers of this study screened the library in *Escherichia coli*, ultimately selecting and culturing 556 colonies for further analysis. This analysis identified 325 unique peptides containing 763 mutations, demonstrating successful synthesis of novel peptides. However, this method for preparing cyclic peptides using the cyanobactin synthesis pathway has not yet been widely adopted because of its low production yield and narrow substrate tolerance.

The Asn/Asp ligase butelase 1, an enzyme capable of catalyzing peptide cyclization, was recently discovered in *Clitoria ternatea* [31]. This enzyme recognizes asparagine or aspartate (Asx) near the C-terminus of a substrate, cleaves the amide bond after Asx, and ligates the N-terminus to the newly formed C-terminus (Figure 3B). Because butelase 1-mediated cyclization requires only Asn-His-Val (NHV) at the C-terminus, the substrate tolerance of this enzyme is extremely high. This advantage makes butelase 1 a good starting point for in vitro/in vivo peptide cyclization. Previously, reported enzymes that can be used for peptide cyclization, such as split inteins (see below), require ~10 amino acids or more for proper reaction [32]. Butelase 1 was shown to cyclize the peptides, kalata B1, conotoxin, thanatin and histatin-3, derived from plants, snails, insects and humans, respectively.

A protein splicing-based peptide cyclization method has also been developed [33]. This technique, called split intein-mediated circular ligation of peptide and proteins (SICLOPPS), catalyzes the ligation of two separate polypeptide chains, called exteins, into one. This is accomplished by transferring a peptide bond between the N-terminal intein domain and C-terminal extein, to the C-terminal intein domain and N-terminal extein. However, to promote conjugation of intramolecular N- and C-terminus, each N- and C-terminal intein domain must be fused at C- and N-termini of an extein (Figure 3C). Using an engineered split intein, these researchers synthesized pseudosterallin F, a plant-derived cyclic peptide tyrosinase inhibitor, in *E. coli*. The engineered split-intein approach has also been used to construct a cyclic peptide library, with the goal of identifying peptide inhibitors of various enzymes [34,35,36]. This peptide library was combined with a bacterial reverse two-hybrid system to screen for inhibitors of protein-protein interactions [37,38]. The split-intein system was further modified to synthesize lariat peptide forms by inhibiting downstream steps after transesterification by mutating Asn, which is responsible for cyclic peptide release [39]. A randomized lariat peptide library was subsequently screened using a yeast two-hybrid system to search for target protein-binding sequences [40]. An artificially designed intein, consensus fast DnaE intein (Cfa), was also recently developed [41]. As its name implies, this enzyme was evolved from consensus sequences of various DnaE inteins. A total of 105 DnaE inteins and 73 theoretically fast inteins were selected for multiple sequence alignment. After selection, the sequences of 73 inteins were used to generate Cfa intein, which has rapid and stable splicing activity. With additional mutations [42], Cfa intein was modified to cyclize eGFP without a dependence on extein sequence. The broad applicability of Cfa intein may be useful for constructing much larger cyclic peptide libraries.

### 3.3. Other Approaches for Peptide Cyclization

Attempts have been made to develop alternative methods for cyclization using endogenous reactive residues. Disulfide bonds are known to stabilize protein structures or protein complexes under oxidative conditions, such as found in the endoplasmic reticulum and extracellular regions [43]. Disulfide bond-mediated cyclization has been widely used to display cyclic peptides on the surface of bacteriophage. A method termed DNA-templated synthesis was developed to synthesize and cyclize peptides containing eight internal scaffold amino acids [44]. This method was used to prepare a library of cyclic tetrapeptides composed mostly of non-proteogenic amino acids. This system was recently expanded to 32 kinds of scaffold amino acids and applied to screen for inhibitory cyclic peptides targeting insulin-degrading enzyme (IDE) [45]. A cyclization method combining the intein system and unnatural amino acids was developed for generating monocyclic peptides (using GyrA intein) or bicyclic peptides (using split intein) [46,47]. One interesting feature of the GyrA-based method is that it is capable of producing side chain-to-tail cyclic peptides. A semi-synthetic method for peptide cyclization has also been reported [48]. In this method, the C-terminal domain of a split intein containing the unnatural amino acid p-acetylphenylalanine (AcF) and random mutations is expressed in *E. coli* using a genetic code expansion method, and the N-terminal domain of the split intein containing a hydroxylamine group and random mutations is chemically synthesized. When N- and C-terminal split inteins are mixed, a splicing reaction and oxime ligation occur between AcF and the hydroxylamine group, generating a cyclic peptide (Figure 3D).

## 4. Screening of Bio-Active Cyclic Peptides

An appropriate screening method is essential for identifying target-binding candidates from large cyclic peptide libraries in a reasonable length of time. Several screening methods have been developed; these can be categorized into in vitro and in vivo methods.

### 4.1. In Vitro Screening

Because there is no chance of interference by intracellular factors with in vitro methods, the primary selection criteria for these methods is binding affinity between the cyclic peptide and target protein. With successive screening steps, peptides with higher affinity can be selected. Using in vitro methods, researchers can also control all relevant variables necessary for appropriate screening, such as concentration of target protein, buffer, and salt conditions. Thus, in vitro methods usually offer a wide, adjustable dynamic range for screening active cyclic peptides [49].

#### 4.1.1. Phage Display

One of the most frequently used in vitro peptide-screening methods is phage display [50]. In general, the phage-display technique uses filamentous bacteriophage to connect genotypes and phenotypes of displayed peptides. Other types of phage can also be used, including T7 phage and Qβ phage [51]. For a peptide to be displayed on a phage particle, it should be connected to the solvent-exposed region of a phage surface protein. The p3 protein of the filamentous bacteriophage, responsible for binding to the F pilus of the host, is most widely used because its N-terminus is exposed to a solvent. Peptides or proteins of various size can therefore be fused to p3 without affecting phage infection or production. Other phage surface proteins, such as p6, p7, p8 and p9, have also successfully been used for phage display [52,53,54,55,56]. After construction and transformation of a randomized peptide library, each peptide can be routinely displayed on the phage surface directly using a phage genome as a library carrier or by transfection of a helper phage in a phagemid system. This method is straightforward, but because the target molecule is often anchored on the hydrophobic surface of a plastic plate or magnetic beads through adsorption, the orientation of the target on the surface depends on the protein’s surface properties. Methods for anchoring target molecules in a fixed orientation using biotin [57] or unnatural amino acids [58] have been established. In this method, library-displaying phages are mixed with an anchored target and incubated for an appropriate time. After binding, unbound phages are washed out and the remaining phages, which are presumably positive clones, are eluted. After repeating this series of steps ~3–5 times, selection-surviving peptides are isolated and identified. The maximum diversity of phage display is ~10^10^ because of limitations in transformation efficiency [59].

#### 4.1.2. mRNA Display

**Figure 4:**
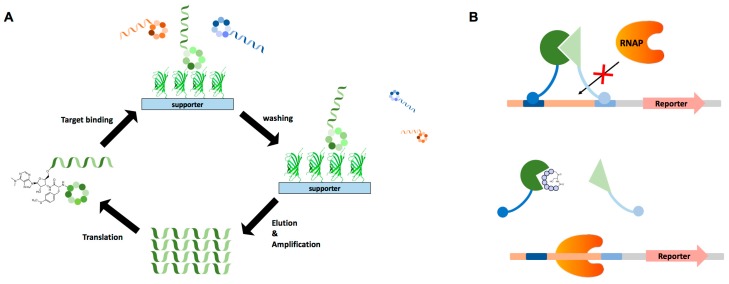
Screening of biologically active cyclic peptides. (**A**) Messenger RNA (mRNA) display-based cyclic peptide library screening. (**B**) Bacterial reverse two-hybrid system used for screening of active cyclic peptides. RNAP: RNA polymerase.

Another powerful in vitro screening method is messenger RNA (mRNA) display. For synthesis of ribosomal polypeptide libraries, mRNA display depends on an in vitro translation system, such as *E. coli* S30 [60], rabbit reticulocyte lysates [61], or the PURE (protein synthesis using recombinant elements) system [62]. The mRNA used for peptide synthesis does not contain a stop codon for translation termination, and instead the translation inhibitor puromycin is conjugated at the 3’ terminus via a flexible linker. When the peptidyl transfer reaction reaches a last codon, the ribosome stalls on the mRNA and puromycin enters the active site of the peptidyl transferase. The newly synthesized peptide is transferred to puromycin and covalently attached to its own mRNA, generating the linkage between genotype and phenotype and enabling in vitro screening of a large pool of potential peptide library. The basic workflow is depicted in Figure 4A. Many uncommon peptides, such as antibacterial peptides and highly hydrophobic or basic peptides, are usually difficult to express and display in a cellular system. However, mRNA display combined with reconstructed translation, as exemplified by the PURE system, is very useful for expressing such uncommon peptides [63]. The Suga group recently developed a modified flexible in vitro translation (FIT) system that allows for genetic incorporation of three non-proteogenic amino acids in addition to 20 basic proteogenic amino acids by dividing codon boxes for valine, glycine and arginine [64]. Using both a FIT-32nt system and flexizyme, an artificial AARS-like ribozyme [65], these researchers were able to incorporate eight different unnatural amino acids into linear and cyclic peptides. In theory, a maximum of 11 different non-proteogenic amino acids can be added to the natural repertoire in a one-pot reaction using this system. In addition, this in vitro display system ensures a higher maximum diversity (~10^13^), since no transformation step is needed [66]. mRNA display has been used extensively to identify many active macrocyclic peptides [67]. For example, the anti-Akt2 macrocyclic peptide, Pakti-L1, isolated by this method was found to have strong inhibitory activity toward Akt2 (half maximum inhibitory concentration, IC_50_ = 110 nM), with only moderate activity towards Akt1 and Akt3d isoforms [68]. mRNA display has also led to the discovery of other macrocyclic peptides with low nanomolar IC_50_ values towards SIRT2 [69] and E6AP [70].

#### 4.1.3. Solid-Phase Peptide Synthesis for Cyclic Peptide Screening

Total chemical synthesis of cyclic peptides is possible through solid-phase peptide synthesis (SPPS) [71]. Since SPPS does not depend on biological factors, amino acids with diverse chemical structures (such as those that are branched, elongated and N-methylated), can theoretically be used as reaction components. Synthesized peptides are anchored on a solid bead and can be modified further as necessary depending on the specific application. Cyclic peptides synthesized by SPPS are used for screening target proteins or molecules labeled with fluorescent dye or other reporters in an interaction-dependent manner. A cyclic peptide library containing specific chemical groups, such as 2-amino-8-hydroxyamino-8-oxooctanoic acid, as a “warhead” was synthesized by SPPS and screened against HDAC, leading to several hits with approximately nanomolar dissociation constant (K_d_) values towards class 1 HDACs [72].

### 4.2. In Vivo Screening

In vivo screening methods rely on living organisms and their translation systems, and are affected by many conditions and factors, including culture conditions, copy number of the gene of interest and expression level of the target protein, among others. The genetic diversity with such screening methods is usually less than 10^10^, because transformation of the library is mandatory. Despite such limitations, many in vivo screening methods have been widely used, since the selected peptides usually have low cytotoxicity. In addition, these methods ensure that the selected candidates inhibit the target, because the selections are often not only interaction-dependent but also activity-dependent.

#### 4.2.1. Yeast Two-Hybrid

One of the most widely used in vivo screening methods is the yeast two-hybrid system. In this interaction-dependent selection method, the target protein is fused to a DNA binding domain and the cyclic peptide library is fused to a transcription-activating domain. When cyclic peptide (bait) interacts with target protein (prey), the resulting expression of a selective marker may be used to screen for cyclic peptide/protein interaction. This method has been used to identify the active cyclic peptides, L2 [39] and TG17 [40], which specifically interact with the bacterial SOS response regulator LexA and Abl kinase, respectively. Interestingly, the cyclic peptide TG17 strongly binds to Abl kinase (K_d_ in the nanomolar range), but its IC_50_ value is quite high (>100 µM), illustrating a clear gap between target-binding ability and cellular inhibitory activity.

#### 4.2.2. Bacterial Reverse Two-Hybrid

The bacterial reverse two-hybrid system (RTHS) is highly useful for screening modulators that inhibit target protein-protein interaction [73]. The RTHS utilizes a bacteriophage repression system [74], in which protein-protein interaction causes suppression of a selection marker, leading to cell death. If effective modulators are present in the cyclic peptide library, they block specific target interactions, causing the selection marker to become expressed and thus enabling cells that harbor the modulator to form colonies (Figure 4B). The RTHS combined with SICLOPPS has been used to screen for inhibitory cyclic peptides targeting specific homo- or heterodimeric protein-protein interactions in *E. coli* [37,38,75]. To date, most of the cyclic peptides screened by RTHS have had relatively weak binding ability (IC_50_ or K_d_ values of ~10 µM) compared with cyclic peptides screened using other affinity-dependent methods, such as phage display and mRNA display.

#### 4.2.3. Protein-Fragment Complementation Assay

Protein-fragment complementation assays (PCAs) using various split reporters have been well documented and can be used for screening of active cyclic peptides. For example, in a split adenylate cyclase system, the interaction between two proteins of interest that are fused to each half of the cyclase molecule causes production of cAMP by the assembled adenylate cyclase, leading to expression of a special selection marker. When *pyrF* (orotidine 5′-phosphoate decarboxylase) is used as a marker, the expressed *pyrF* produces the toxic uracil analog 5-fluorouracil from the nontoxic compound 5-fluoroortic acid (5-FOA), ultimately leading to cell death. If an active cyclic peptide effectively inhibits this protein–protein interaction, expression of the *pyrF* gene is blocked; as a result, cells can form colonies in the presence 5-FOA [76]. However, with some rare exceptions, including luciferase-based PCAs, the assembly and disassembly of the components of many split reporters are not reversible and fragment pairs often form reporter complexes spontaneously without the induction of protein-protein interactions [77,78,79]. One such example is fluorescent protein-based PCAs, which often generate high background fluorescence owing to spontaneous assembly. Therefore, to date, most PCA methods have been used to detect protein–protein interactions, and not to screen for modulators of such interactions.

#### 4.2.4. Other In Vivo Screening Approaches

In addition to targeting specific interactions between two proteins of interest, methods to screen for cyclic peptides that inhibit specific cellular enzymes have been established. DNA adenine methyltransferase (DAM methylase), which regulates the methylation state of bacterial chromosomes, is an important target for antibiotics development. To screen for inhibitors of the DAM methylase of *E. coli*, transposase-based approach was established [35]. If cyclic peptides in the library are able to inhibit DAM methylase, transposase can bind and transfer its unmethylated substrates including a chloramphenicol acetyltransferase gene and the cyclic peptide library SICLOPPS to the F plasmid. F plasmids containing transposons are transferred to transposase-lacking bacteria via conjugation, leading to colony formation on chloramphenicol-selective plates. After screening, the authors of this study obtained several active cyclic peptides. The selected cyclic peptide (SGWYVRNM) exhibited an inhibitory effect on DAM methylase comparable to that of the previously known small molecule inhibitor sinefungin, whereas a linearized peptide with the same sequence did not. The importance of cyclization is also illustrated by active cyclic peptides carrying the unnatural amino acid, p-benzoylphenylalanine, which shows inhibitory activity toward HIV protease only in circular form. An active cyclic peptide that inhibits the protein degradation pathway was also isolated [36]. In this application, green fluorescent protein (GFP) fused to an ssrA-tag sequence that is recognized for degradation by ClpXP protease was transformed into *E. coli* together with a cyclic peptide library SICLOPPS. Active cyclic peptides with inhibitory activity against ClpXP protease lead to accumulation of the GFP-ssrA fusion protein, and thus emit a strong fluorescent signal. As expected, the cyclic form was much more potent than the linear form. Finally, active cyclic peptides with the ability to neutralize neural toxicity have been isolated using a cyclic peptide library SICLOPPS expressed in a yeast strain that mimics the neural toxicity caused by aggregation of α-synuclein [80]. Interestingly, the cyclic peptides selected were able to reduce the toxicity of α-synuclein in *Caenorhabditis elegans* dopaminergic neurons.

## 5. Active Cyclic Peptides as Inhibitors and Molecular Probes

**Figure 5:**
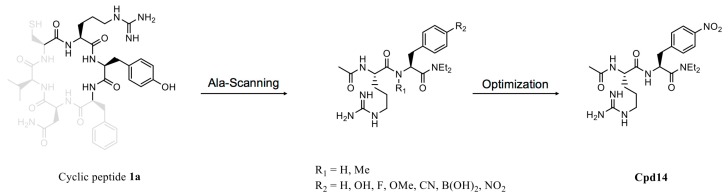
Optimization of active cyclic peptide. Ala-scanning of cyclic peptide 1a identified two essential amino acids, arginine and tyrosine, for target binding. Subsequent optimization led to a more potent dipeptide, Cpd14 (compound 14).

As previously noted, screening for active cyclic peptides based on their target-binding ability does not ensure cellular inhibitory activity, emphasizing the importance of further derivatization and optimization in the development of efficient inhibitors from cyclic peptide hits. To date, there have been few successful cases of such derivatization and optimization from active cyclic peptide hits. In one such rare case, an in vivo method was used to screen a cyclic peptide library for an inhibitor that disrupts homodimeric interactions of aminoimidazole-4-carboxamide ribonucleotide (AICAR) transformylase, which catalyzes the last two steps of de novo purine synthesis [37]. This screen yielded the active cyclic peptide RYFNVC (peptide 1a), which exhibited a K_i_ value of 17 µM in circular form (Figure 5). An Ala-scanning analysis revealed that arginine and tyrosine played an important role in binding to the target protein [81]. Subsequent optimization of the hit led to more potent dipeptides, including Cpd14 (compound 14), which possessed the lowest K_i_ value (0.685 µM) and effectively inhibited proliferation of MCF-7 breast cancer cells. Cpd14 was also used to study metabolic disorders in a mouse model [82]. In high-fat-diet-fed mice treated with Cpd14, glucose levels in the blood rapidly decreased, an effect not observed in regular chow-fed mice. These results illustrate the potential of screened active cyclic peptides to serve as useful leads for multiple biomedical purposes.

Most studies on cyclic peptides have focused on the possible role of these peptides as inhibitors. However, the high binding affinity and selectivity of cyclic peptides towards a target molecule can be expanded to include use as a molecular probe. Somatostatin, a naturally occurring cyclic peptide hormone, interacts with somatostatin receptors, which are overexpressed in many cancers, including gliomas, neuroendocrine tumors, breast cancer, and small-cell lung cancer [83]. Unfortunately, unmodified somatostatin cannot be used as a probe, owing to its low serum stability (half-life < 3 min) [84]. Accordingly, somatostatin was modified to develop cyclic peptides with higher resistance to in vivo proteases. The ^111^In-labeled cyclic octapeptide octreotide, fCFwKTCT-ol (where lower-case letters represent D-amino acids) is the first FDA-approved peptide-based imaging agent for neuroendocrine tumors. Several ^111^In-labeled octreotide variants and derivatives have subsequently been synthesized for possible clinical use [85]. The Arg-Gly-Asp (RGD) tripeptide also provides a good starting point for probe development, since it selectively binds to the α_v_β_3_ receptor. RGD-containing cyclic peptides have been labeled with various radioisotopes, including ^18^F, ^64^Cu, ^68^Ga and ^99m^Tc, to generate a new class of probes [86,87,88,89].

## 6. Conclusions

Because of their special functional features, including unique selectivity, versatility and structural stability, cyclic peptides have attracted increasing attention as a promising alternative to currently used small molecule and macromolecule pharmaceutical scaffolds. Many natural cyclic peptides have been modified further to develop biomedically useful compounds. Recent notable progress in peptide cyclization and screening technologies will expand the use of biologically or organically designed and synthesized cyclic peptides for therapeutic purposes. Rapid development of computational tools, including in silico-guided peptide library generation, will further accelerate cyclic peptide-based drug development. The selective and strong binding ability of cyclic peptides towards specific targets will find diverse important applications, including as excellent tools for probing specific proteins or metabolites in vivo. In addition, cyclic peptide-based chemical structures will find broader future use in various fields, including as building blocks for macromolecules.
